# A Data Self-Calibration Method Based on High-Density Parallel Plate Diffuse Optical Tomography for Breast Cancer Imaging

**DOI:** 10.3389/fonc.2021.786289

**Published:** 2021-12-21

**Authors:** Xin Wang, Rui Hu, Yirong Wang, Qiang Yan, Yihan Wang, Fei Kang, Shouping Zhu

**Affiliations:** ^1^ School of Life Science and Technology, Xidian University, Xi’an, China; ^2^ Engineering Research Center of Molecular and Neuro Imaging of Ministry of Education, Xi’an, China; ^3^ Department of Nuclear Medicine, Xijing Hospital, Fourth Military Medical University, Xi’an, China

**Keywords:** diffuse optical tomography (DOT), data calibration, medical optics instrumentation, image reconstruction, breast cancer

## Abstract

When performing the diffuse optical tomography (DOT) of the breast, the mismatch between the forward model and the experimental conditions will significantly hinder the reconstruction accuracy. Therefore, the reference measurement is commonly used to calibrate the measured data before the reconstruction. However, it is complicated to customize corresponding reference phantoms based on the breast shape and background optical parameters of different subjects in clinical trials. Furthermore, although high-density (HD) DOT configuration has been proven to improve imaging quality, a large number of source-detector (SD) pairs also increase the difficulty of multi-channel correction. To enhance the applicability of the breast DOT, a data self-calibration method based on an HD parallel-plate DOT system is proposed in this paper to replace the conventional relative measurement on a reference phantom. The reference predicted data can be constructed directly from the measurement data with the support of the HD-DOT system, which has nearly a hundred sets of measurements at each SD distance. The proposed scheme has been validated by Monte Carlo (MC) simulation, breast-size phantom experiments, and clinical trials, exhibiting the feasibility in ensuring the quality of the DOT reconstruction while effectively reducing the complexity associated with relative measurements on reference phantoms.

## 1 Introduction

Diffuse optical tomography (DOT) is a highly specific functional imaging modality for breast cancer imaging, which can offer a low-cost, non-invasive, and portable alternative technology to magnetic resonance imaging (MRI), positron emission tomography (PET), and digital breast tomosynthesis (DBT) (1–5). Photon propagation in breast tissue can be described by the diffusion equation (DE), and the absorption distribution in the tissue is obtained by solving the inverse problem. Based on multi-wavelength measurements and spectral unmixing, the concentration distribution of chromophores in breast tissue can be further calculated (6, 7). Tumor tissue usually exhibits increased blood vessel density and decreased oxygen content, which leads to a higher total hemoglobin concentration and lower blood oxygen saturation. Therefore, optical contrast can be used to characterize the disease (8). However, since biological tissue is the turbid and highly scattering media, the quality of the reconstructed DOT image is unsatisfactory (9).

Several methods have been proposed to improve the quality of the DOT reconstruction. On the one hand, some groups have proposed modified regularization algorithms for optimizing the inverse problem (10–13). The other groups have employed information on the size, shape, and depth of the lesion obtained with high-resolution imaging equipment to guide DOT reconstruction (14, 15). On the other hand, many researchers are working on improving the imaging system. High-density (HD) settings (measurements typically obtained using arrays with the nearest-neighbor source-detector (SD) distance <1.5 cm) have become one of the trends in system updates. It has been proven to have advantages in enhancing imaging resolution (16). For breast imaging, the HD-DOT system is capable of providing high sensitivity, large dynamic range, and large imaging field of view (17–19). Although both of the mentioned methods help to improve the imaging quality, the experimental accuracy will be affected by system errors, including noise and the poor coupling between the SD pairs and the imaging subjects, etc. Especially for HD-DOT systems, the inconsistency between multi-channels is significant due to the large number of the SD pairs, and these factors will have a greater impact on the measurement data (20–23). Therefore, the calibration of experimental data is also critical.

The measured data of DOT are conventionally calibrated by reference measurement. This approach usually needs to use a reference phantom with the same background optical parameter and size as the task (24). However, it is complicated to customize corresponding reference phantoms based on the exact tissue morphology (e.g., the breast shape) of different subjects in clinical trials. Several studies have been proposed to utilize the contralateral healthy breast to calibrate the measurement from the tumor-bearing breast. Althobaiti et al. have introduced an automated preprocessing method, which requires continuous correlation analysis of multi-wavelength data (25, 26). Li et al. have also proposed an outlier removal algorithm, which needs to determine an appropriate threshold (27). Nevertheless, the differences between the size and the detection conditions of the two breasts can also introduce artifacts (28).

In practical clinical application of the breast DOT, to address the defects and limitations of the reference measurement in the above analysis, a data self-calibration method based on an HD parallel-plate DOT system is proposed in this paper, aiming to replace the conventional relative measurement on a reference phantom. Thanks to the geometric symmetry of the HD-DOT system (29), each SD distance corresponds to many different SD pairs at different locations. For the diffuse optical measurement of the large-size homogeneous turbid media (e.g., normal soft tissue), different SD pairs with the same SD distance in non-boundary regions have similar outgoing light intensity. In this case, the measured values under the same SD distance are approximately the same value. Considering the limited size of the tumor relative to the whole breast, it is assumed that the maximum value of the data obtained from SD pairs at different locations at the same SD distance is the measurement under the condition of hardly passing through the absorption heterogeneous region, such as the lesion tissue. At this time, this measurement value is basically the same as that obtained at the same SD distance on the reference homogeneous phantom. Based on the above analysis, the reference prediction data for one SD distance can be constructed directly from the measurement data according to the maximum value of a set of measurements in the non-boundary region with the same SD distance. The proposed approach is validated by Monte Carlo (MC) simulation and is verified by breast-size phantom experiments and clinical trials on a self-build HD-DOT system, exhibiting its feasibility in DOT reconstruction while effectively reducing the complexity associated with relative measurements on reference phantoms.

## 2 Methods

### 2.1 Forward Model

Near-infrared light will be absorbed and scattered in tissue. The forward transmission process of light in tissue is often described by the diffusion equation (DE) (30) as shown in Eq. (1)


(1) $$-\nabla \cdot [\kappa \nabla \Phi (r)]+\mu_{a}(r)c\Phi (r)=q_{0}(r),(r\in \Omega )$$

where *Ω* is the tissue region, and *Φ*(*r*) represents the photon density at position *r*. *q*_0_ is the source term. $\kappa =c/[3*(\mu_{a}+\mu_{s}^{\prime })]$ is the diffusion coefficient, and *c* is the propagation speed of light in tissue. *μ_a_* represents the absorption coefficient, and $\mu_{s}^{\prime }$ represents the reduced scattering coefficient.

The boundary condition can be expressed by Eq. (2)


(2) $$c\Phi (r)+2\kappa \zeta {\hat{s}}_{n}\cdot \nabla \Phi (r)=0,(r\in \partial \Omega )$$

where ζ = (1 + *R_f_*)/(1 – *R_f_*), and *R_f_* is the diffuse transmission internal reflection coefficient. ${\hat{s}}_{n}$ is the surface outward normal unit vector.

The output light flux detected on the surface of the tissue is


(3) $$\Gamma (r_{s}^{i},r_{d}^{j})=-\kappa {\hat{s}}_{n}\cdot \nabla \Phi (r_{s}^{i},r_{d}^{j}),(r_{s}^{i},r_{d}^{j}\in \partial \Omega ;\;i=1,2,\cdots ,I;\;j=1,2,\cdots ,J)$$

where ∂*Ω* represents the surface of the tissue. $r_{s}^{i},(i=1,2\cdots I)$ represents the *i*-th source position with the total number of *I* $r_{d}^{j},(j=1,2,\cdots ,J)$ represents the *j*-th detector position with the total number of *J*.

### 2.2 Inverse Problem

In this paper, We utilize the finite-element-methods (FEM) based software package NIRFAST (31) to solve the continuous wave (CW) DOT inverse problem (32), which can be described as Eq. (4)


(4) $$\mu_{a}(r)=F^{-1}[\Gamma (r_{s}^{i},r_{d}^{j})],(r\in \Omega ;\;r_{s}^{i},r_{d}^{j}\in \partial \Omega )$$

where *F* is forward model. Since the inverse problem is highly ill-posed, Tikhonov regularization is used to constrain the reconstruction, as shown in Eq. (5)


(5) $$\mu_{a}(r)=\arg\min\left\{{∥\Gamma (r_{s}^{i},r_{d}^{j})-F(\mu_{a}(r))∥}^{2}+\lambda {∥\mu_{a}(r)∥}^{2}\right\},\left(r\in \Omega ;\;r_{s}^{i},r_{d}^{j}\in \partial \Omega \right)$$

where *λ* is the regularization parameter.

### 2.3 Data Calibration Method

#### 2.3.1 Reference Phantom Calibration Method

The conventional relative measurements using a reference phantom require two scans, including a scan of the task and a scan of a reference homogeneous phantom with the same shape and background optical parameters as the task. The specific steps are as follows:

(1) The measured data of the task can be expressed as $\Gamma^{tsk}(r_{s}^{i},r_{d}^{j})$ .

(2) Similarly, the measured data of the reference phantom can be expressed as $\Gamma^{ref}(r_{s}^{i},r_{d}^{j})$ .

(3) Use the NIRFAST software to generate an optical calculation model of the homogeneous phantom with the same shape and background optical parameters as the task, the simulated forward data $\Gamma^{pre}(r_{s}^{i},r_{d}^{j})$ can be obtained.

(4) The reference phantom calibration method can be expressed by Eq. (6)


(6) $$\Gamma^{*}(r_{s}^{i},r_{d}^{j})=\frac{\Gamma^{tsk}(r_{s}^{i},r_{d}^{j})}{\Gamma^{ref}(r_{s}^{i},r_{d}^{j})}\Gamma^{pre}(r_{s}^{i},r_{d}^{j}),(i=1,2,\cdots ,I;\;j=1,2,\cdots ,J)$$

where *Γ* ^∗^ is the calibrated data used for reconstruction. Since the size of the task phantom is known, it is easy to implement reference measurement in phantom experiments. However, the reference phantom for each patient is difficult to make on site. Therefore, a calibration method without using the reference homogeneous phantom is needed.

#### 2.3.2 Data Self-Calibration Method

**Figure 1:**
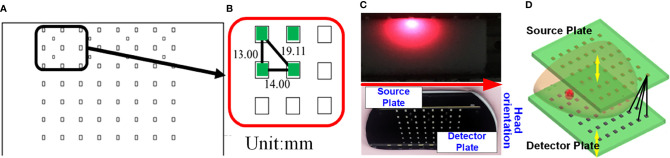
Schematic of the source and detector plates of the DOT system. **(A)** Arrangement of the light source array (or optical sensor array). **(B)** The row and column spacing between the LEDs (or PDs). **(C)** Photograph of the source plate and the detector plate. **(D)** The black lines indicating the different SD pairs.

The DOT imager used in this work is a self-built fiber-free high-density parallel plate CW system based on multi-wavelength light-emitting diodes (LEDs) and high-sensitivity photodiodes (PDs). The LED contains three wavelengths: 660 nm, 750 nm, and 840 nm, and both the sources and the detectors are surface-mount devices. The arrangement of the optical sensor array is completely consistent with that of the LED array, that is, each optical sensor is in the mirror position of the opposite LED. The sources and the detectors are arranged in 7 rows and 8 columns. The row spacing is 13 mm, and the column spacing is 14 mm. **Figures 1A–C** illustrate the structure of the source and detector plates. **Figure 1D** presents the different SD pairs used to perform measurements.

The implementation steps of the data self-calibration method proposed in this paper are as follows.

(1) The measured data of the task obtained by the DOT system can be expressed as $\Gamma^{tsk}(r_{s}^{i},r_{d}^{j})$ .

(2) Calculate the SD distance *d_ij_* according to different SD pairs:


(7) $$d_{ij}=\left|r_{s}^{i}-r_{d}^{j}\right|,(i=1,2,\cdots ,I;\;j=1,2,\cdots ,J)$$

where *d_ij_* represents the Euclidean distance between the *i*-th source and the *j*-th detector.

(3) Based on the geometric symmetry of the system’s SD arrangement, there will be cases where different SD pairs have the same SD distance. Traversing all SD pairs, a total of *K* different SD distances can be obtained, i.e., *d_ij_* ∈{*d*_1_,···,*d_k_*, ···,*d_K_*}. Group the measured data with the same SD distance (*d_k_*) into the same set and record it as *Ω_k_*, (*k* ∈[1,*K*]):


(8) $$\Omega_{k}=\left\{\Gamma^{tsk}(r_{s}^{i},r_{d}^{j})|d_{ij}=d_{k}\right\},\;\left(i=1,2,\cdots ,I;\;j=1,2,\cdots ,J\right)$$

(4) The maximum measurement value in *Ω_k_* is selected and expressed as $\Gamma_{k}^{\max}$ ,


(9) $$\Gamma_{k}^{\max}=\max\{\Omega_{k}\},\;k\in [1,K]$$

(5) Set the corresponding maximum measurement value when the SD distance is *d_k_* as the estimated measurement value of the constructed ‘virtual homogeneous phantom’ at the same SD distance (*d_k_*), which is expressed as


(10) $$\Gamma^{est}(r_{s}^{i},r_{d}^{j})=\Gamma_{k}^{\max}$$

where *d_ij_* = *d_k_*, (*i* = 1,2,···,*I*; *j* = 1,2,···,*J*). The measured data of the ‘virtual homogeneous phantom’ under all SD pairs can be further obtained as $\Gamma^{est}(r_{s}^{i},r_{d}^{j}),\;(d_{ij}\in \{d_{1},\cdots ,d_{k},\cdots ,d_{K}\};\;i=1,2,\cdots ,I;\;j=1,2,\cdots ,J)$ .

(6) Replacing $\Gamma^{ref}(r_{s}^{i},r_{d}^{j})$ in Eq. (6) by $\Gamma^{est}(r_{s}^{i},r_{d}^{j})$ , which in turn leads to the expression for the data self-calibration strategy:


(11) $$\Gamma^{**}(r_{s}^{i},r_{d}^{j})=\frac{\Gamma^{tsk}(r_{s}^{i},r_{d}^{j})}{\Gamma^{est}(r_{s}^{i},r_{d}^{j})}\Gamma^{pre}(r_{s}^{i},r_{d}^{j}),\;\left(i=1,2,\cdots ,I;\;j=1,2,\cdots ,J\right)$$

It should be noted that some SD pairs need to be removed before applying the data self-calibration method. The criteria for excluding SD pairs in this work are: (1) the SD pairs that are not covered by the imaging object needs to be eliminated, and (2) the SD pairs with SD distance greater than 110 mm should be eliminated.

## 3 Experiments

### 3.1 Monte Carlo Simulation Experiment

We first used MC simulation to verify the feasibility of the proposed method. All simulations were performed with the MCX simulator (33). A three-dimensional breast-size domain with a thickness of 44 mm and with a parabolic contour matching the shape of a healthy female breast was used to generate simulated CW measurements. The volume of the domain is 79mm × 130mm × 44mm, and the size of a single pixel is 1mm × 1mm × 1mm. The optical properties of the breast tissue were set to *μ_a_* = 0.0051mm^-1^, and $\mu_{s}^{\prime }$ = 1.090mm^-1^ at 760 nm (34). In bio-tissue imaging experiment, polyoxymethylene (POM) was often employed to make the reference phantom, and its optical properties were determined to be *μ_a_* = 0.0038mm^-1^, and $\mu_{s}^{\prime }$ = 0.9380mm^-1^ at 670 nm (35). Therefore, in this experiment, the optical properties of the simulation volume of interest (VOI) were set to *μ_a_* = 0.004mm^-1^, $\mu_{s}^{\prime }$ = 1mm^-1^, *g*=0.9, and *n*=1.33, respectively.

**Figure 2:**
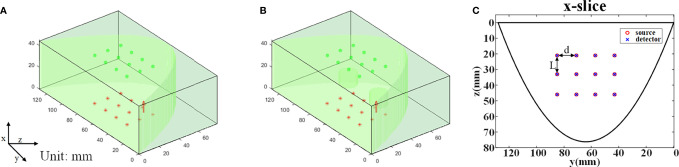
MC simulation settings. **(A)** The reference phantom. **(B)** The task phantom. **(C)** The arrangement of the light sources and the detectors, *L*=13 mm, *d*=14 mm.

A homogeneous model was simulated to generate reference phantom data (**Figure 2A**). Another model was simulated to generate task phantom data, which had two cylindrical targets with a height of 10 mm and a diameter of 15 mm (**Figure 2B**). The horizontal distance between the two targets is 40 mm and the center coordinates of the targets are (17, 45, 42), (17, 85, 42) (unit: mm), respectively. The absorption coefficient of the two targets is three times that of the background, i.e., *μ_a_* = 0.012mm^-1^, and other optical parameters of the targets are the same as the background.

The arrangement of the source array is completely consistent with that of the detector array, i.e., 3 rows and 4 columns (**Figure 2C**). The row spacing *d* is 13 mm, and the column spacing *L* is 14 mm. The light sources are placed on the top plane (x=0 mm) and the detectors are placed on the bottom plane (x=44 mm). The type of light source is ‘cone’ with a 120° divergence angle and the diameter of the detector is 1.5 mm. The total number of photons set in the simulation is 2 x 10^9^.

### 3.2 Phantom Experiment

**Figure 3:**
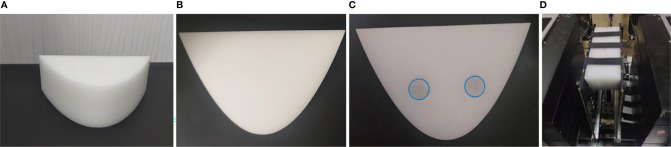
Phantom experiment settings. **(A)** The reference phantom. **(B, C)** The task phantom. **(D)** Experimental device.

To further verify the effectiveness of the proposed scheme, a series of phantoms made of POM with the same size as the phantom in MC simulation were used to obtain measured data. The thickness of the reference phantom is 44 mm, as shown in **Figure 3A**. The task phantom is composed of two POM blocks with a thickness of 22 mm, one of which is completely homogeneous (**Figure 3B**) and the other has two cylindrical holes (**Figure 3C**). The size of the phantom is detailed in the previous section.

The two cylindrical holes in the POM phantom were filled with the optical absorption target, as shown in **Figure 3C**. The target was made of a combination of intralipid (20%) and India ink. The concentration of intralipid (20%) and Indian ink required for the experiment can be determined according to the empirical formula and the absorbance measured by the spectrometer, respectively (36). The optical properties of both targets were *μ_a_* = 0.03mm^-1^, $\mu_{s}^{\prime }$ = 1mm^-1^. 2% agar powder was used to solidify the above mixed solution.

In this experiment, we first placed the phantom in the center of the SD plates and adjusted the distance between the double plates to 44 mm (**Figure 3D**), and then the data collection had been performed in a dark room. The reference phantom and the task phantom were measured sequentially, and the total scanning time was within 6 minutes.

### 3.3 Clinical Trials

The subject was a 60-years-old postmenopausal female with a body mass index (BMI) of 23 kg/m^2^ undergoing endocrine therapy for cancer of the right breast. This retrospective study was approved by the Ethics Committee of Xijing Hospital (Approval No. KY20212008-F-1). The patient was diagnosed by core biopsy revealing invasive micropapillary carcinoma in the right breast. The molecular subtype of the tumor was luminal B (ER-positive and/or PR-positive, HER2-positive or HER2-negative with high Ki-67≥20 and higher grade (II or III)) (37). DOT and PET measurements were performed on the patient before the treatment, and these measurements were made in the Nuclear Medicine Department of Xijing Hospital. The patient was injected with 432.9 MBq ^18^F-FDG and scanned after 70 min post injection. During the measurement, the subject lay prone on the cushioned bed and placed the breast in the center of the SD plates by adjusting the body position. The source plate and detector plate were controlled to slightly squeeze the breast, and the distance between the two plates was 57 mm. Then the DOT and PET scans were performed simultaneously on the patient’s breast. The whole measurement was carried out in a dark room, and the total acquisition time was less than 15 minutes.

## 4 Results

### 4.1 Monte Carlo Simulation Experiment

We first evaluated the difference between the estimated virtual reference measurement data and the reference phantom data in MC simulation using the relative error as Eq. (12):


(12) $$error=\frac{1}{I\times J}\sum_{i=1}^{I}\sum_{j=1}^{J}\frac{\left|\Gamma^{est}(r_{s}^{i},r_{d}^{j})-\Gamma^{ref}(r_{s}^{i},r_{d}^{j})\right|}{\Gamma^{ref}(r_{s}^{i},r_{d}^{j})},\;\left(i=1,2,\cdots ,I;\;j=1,2,\cdots ,J\right)$$

**Figure 4:**
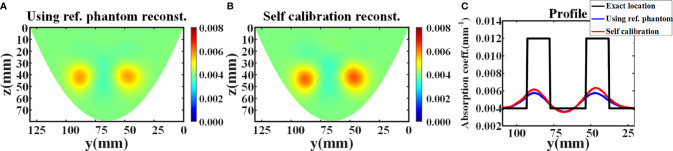
Reconstruction results of the simulation experiment. **(A, B)** The x-slice (x=17 mm) images of reconstructed absorption coefficient distribution using reference measurement and data self-calibration method, respectively. **(C)** The corresponding absorption profiles through the center of two inclusions along the y-axis (z=42 mm).

In this experiment, the average relative error is 4.43%. Then, the DOT reconstruction was carried out. The number of nodes in the reconstructed mesh is 22,134, and the number of the tetrahedral elements is 114,603. The number of iterations is 6, and a fixed *λ* of 10 is selected for each iteration. **Figure 4A** illustrates the absorption coefficient image recovered using reference phantom measurement. The maximum reconstructed absorption coefficient of the two targets are 0.005764 mm^-1^ and 0.005760 mm^-1^ respectively, and the contrast ratio of the two targets is 1.0007:1. **Figure 4B** shows the absorption coefficient image recovered using the data self-calibration method. The reconstructed values of the two targets are 0.006159 mm^-1^ and 0.006347 mm^-1^ respectively, and the reconstructed contrast ratio of the two targets is 0.9704:1. The intensity profiles of the reconstructed absorption images (**Figures 4A, B** **)** recovered using the two methods are shown in **Figure 4C**. **Table 1** displays the full width at half maxima (FWHM) of the intensity profile of each target. All results demonstrate that the data self-calibration scheme has high reconstruction accuracy.

**Table 1:** The FWHM of the reconstructed absorption intensity profile of each target in MC simulation.

	Target1 FWHM (mm)	Target2 FWHM (mm)
Exact	15	15
Using reference phantom	15.5	17.5
Self-calibration method	13	13

### 4.2 Phantom Experiment

**Figure 5:**
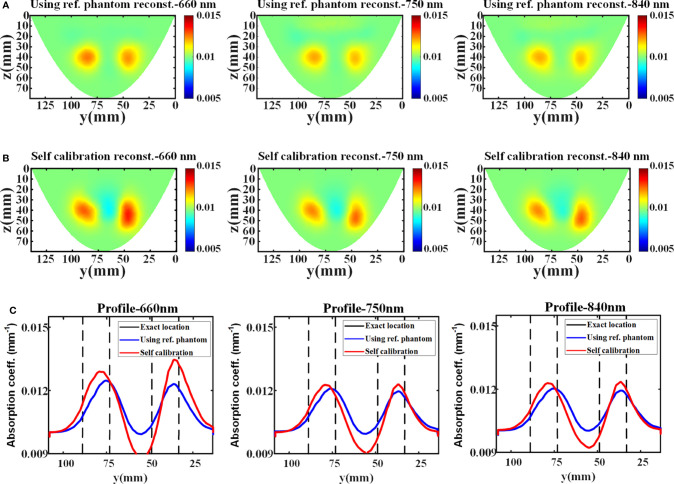
Reconstruction results of the phantom experiment. **(A, B)** The x-slice (x=15 mm) images of reconstructed absorption coefficients with different wavelengths using reference measurement and data self-calibration method, respectively. **(C)** The corresponding absorption profiles through the center of two targets along the y-axis (z=42 mm).

In phantom experiment, the average relative errors of the data measured directly from the reference phantom and estimated by the proposed data self-calibration method are 12.03%, 7.66%, and 6.11% at 660 nm, 750 nm, and 840 nm, respectively. In DOT reconstruction, the FEM mesh contains 76,504 linear tetrahedral elements that are joined at 15,258 nodes. **Figures 5A, B** show the reconstructed absorption images with different wavelengths using the conventional and the proposed method, respectively. The results indicate that the distribution of absorption coefficients recovered by the self-calibration method is similar to the reconstruction result using the reference phantom, and the two methods have similar reconstruction resolution (**Figure 5C**).

**Table 2** shows the reconstructed absorption contrast between two targets in the phantom experiment. The maximum reconstructed absorption coefficients of target 1 at different wavelengths recovered using reference measurement are 0.01246, 0.01204, and 0.01200 (unit: mm^-1^), at 660 nm, 750 nm, and 840 nm, respectively. The maximum reconstructed absorption coefficients of target 2 at different wavelengths are 0.01229, 0.01190, and 0.01190 (unit: mm^-1^), respectively. For comparison, the maximum reconstructed absorption coefficients of target 1 using the data self-calibration method are 0.01279, 0.01239, and 0.01237 (unit: mm^-1^), at 660 nm, 750 nm, and 840 nm, respectively, and the reconstructed values of target 2 at different wavelengths are 0.01345, 0.01242, and 0.01242 (unit: mm^-1^), respectively. The contrast of the quantitative reconstructions (target 1: target 2) obtained by the two methods shows consistency. All results of the phantom experiment further verify the feasibility of the data self-calibration scheme.

**Table 2:** The contrast of the quantitative reconstructions (target 1: target 2) in phantom experiments.

	660 nm	750 nm	840 nm
Exact	1:1	1:1	1:1
Using reference phantom	1.0138:1	1.0118:1	1.0084:1
Self-calibration method	0.9509:1	0.9984:1	0.9960:1

### 4.3 Clinical Trials

**Figure 6:**
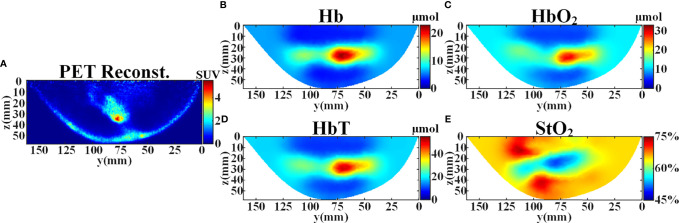
Reconstruction results of the patient’s right breast in y-z plane at x=28 mm before the treatment. **(A)** SUV distribution reconstructed from PET. **(B)** Reconstructed concentration distribution of deoxyhemoglobin (Hb). **(C)** Reconstructed concentration distribution of oxyhemoglobin (HbO_2_). **(D)** Reconstructed concentration distribution of total hemoglobin (HbT). **(E)** Reconstructed oxygen saturation distribution (StO_2_).

PET image is used as cross validation for evaluating the DOT reconstruction. PET and DOT measurements are performed in the same body position, and it is easy to extract the contour of breast tissue from the reconstructed PET image. Therefore, we can generate the FEM mesh of the breast employing the obtained contour and the set SD distance. According to the reconstructed PET image (**Figure 6A**), the tumor is located in the center of the right breast with maximum standard uptake value (SUV) of 5.15. For DOT measurement, the background optical parameters at 750 nm and 840 nm were set as (*μ_a_* = 0.005 mm^-1^, $\mu_{s}^{\prime }$ = 1.09 mm^-1^) and (*μ*_a_ = 0.0055 mm^-1^, $\mu_{s}^{\prime }$ = 1.03 mm^-1^), respectively. The FEM mesh contains 60,196 linear tetrahedral elements that are joined at 12,360 nodes. **Figures 6B–E** illustrate the concentration images of deoxyhemoglobin (Hb), oxyhemoglobin (HbO_2_), total hemoglobin (HbT), and oxygen saturation (StO_2_). The averaged tumor to background (T/B) contrast is calculated to be 3.09× for Hb, 2.13× for HbO_2_, 2.46× for HbT, and 0.87× for StO_2_. David et al. have found that increases in HbT and $\mu_{s}^{\prime }$ contrast showed correspondence with similar high-FDG regions in the PET images, which demonstrates that DOT is indeed sensitive to the local metabolism and may provide information complementary to PET (38). It can also be seen from the results that the high-FDG region in the PET result corresponds to low blood oxygen saturation and high hemoglobin concentration in the DOT result, which is consistent with the literature reports.

## 5 Discussion

**Figure 7:**
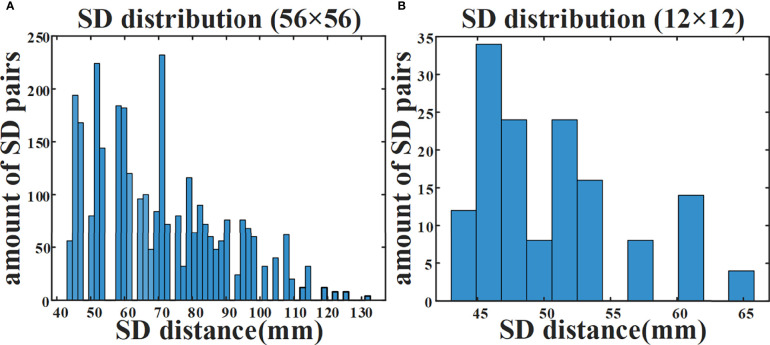
Amount of available SD pairs corresponding to different SD distances. **(A)** Distribution of 3136 SD pairs available to the DOT system. **(B)** Distribution of 144 SD pairs in simulation experiment.

We have introduced a data self-calibration method for high-density parallel plate DOT. Since the calibration is performed directly from the measured data without using the reference phantom, the measurement complexity is effectively reduced. The system contains a total of 56×56 = 3136 available SD pairs. **Figure 7A** illustrates the number of available SD pairs corresponding to each SD distance when double plate spacing is 44 mm. In the simulation experiment, we used 12×12 = 144 SD pairs for DOT measurement, and **Figure 7B** shows the distribution of different SD distances corresponding to these 144 SD pairs.

**Figure 8:**
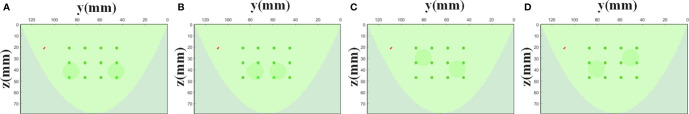
Simulation settings where two targets are placed in different positions. The * represents the setting of the above MC simulation experiment in section 4.1. **(A)** Position*. **(B)** Position1. **(C)** Position2. **(D)** Position3.

**Figure 9:**
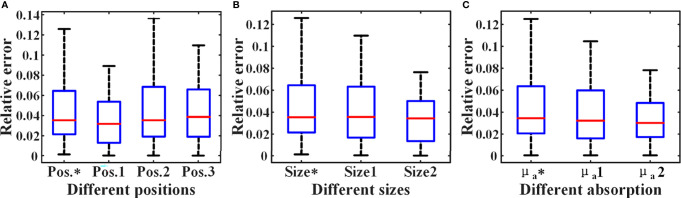
Relative errors under different simulation settings. The * represents the setting of the above MC simulation experiment in section 4.1. **(A)** Relative errors of different positions. **(B)** Relative errors of different sizes. **(C)** Relative errors of different absorption coefficients.

When all 3136 SD pairs are used, the HD-DOT system has nearly a hundred sets of measurements at each SD distance (**Figure 7A**). This provides support for the estimation of the ‘virtual reference measurement’ for each SD distance. Even if only a small number of SD pairs are employed in the simulation experiment, there are more than five measured values from different SD pairs with the same SD distance (**Figure 7B**). The experimental results have verified the effectiveness of the proposed data self-calibration method. In this section, we conduct a series of experimental analyses to test the robustness of the proposed method. First, we analyze whether the targets at different positions will affect the accuracy of the estimated virtual reference data. The size of the two cylindrical targets remains unchanged, with a diameter of 15 mm and a height of 10 mm. **Figures 8A–D** show four different positions. **Figure 9A** exhibits the relative error between the virtual and the real reference phantom data. The average relative errors under the four positions range from 3.50% to 4.74%. The results demonstrate that the position of the targets has little effect on the proposed method.

**Figure 10:**
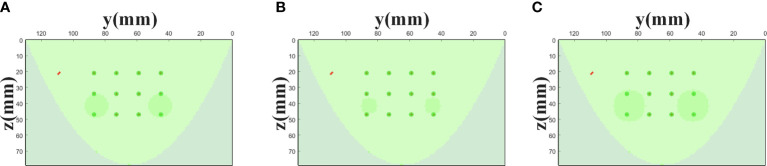
Simulation settings with different target sizes. The * represents the setting of the above MC simulation experiment in section 4.1. **(A)** Size* (R=7.5 mm). **(B)** Size1 (R=5 mm). **(C)** Size2 (R=10 mm).

Then, we analyze the influence of the targets of different sizes on the proposed method. The height of the two cylindrical targets is 10 mm, and the center coordinates are (17,45,42) and (17,85,42) (unit: mm), respectively. As shown in **Figures 10A–C**, we set three different target sizes.

**Figure 9B** shows the relative errors for three different target size settings in **Figure 10**, and the average errors are 4.43%, 4.16%, 3.32%, respectively. The result indicates that the size of the targets has little effect on the accuracy of the proposed strategy. In addition, we have analyzed the impact of target absorption on the method. The radius of the two cylindrical targets is 7.5 mm, and the height is 10 mm. The center coordinates are (17, 45, 42) and (17, 85, 42) (Unit: mm), respectively. We set the absorption coefficients of the two targets to be 2-4 times that of the background (*μ*_a_ = 0.004mm^-1^), and the relative errors in all cases are shown in **Figure 9C**. In summary, the generalization of the data self-correction method is verified.

**Figure 11:**
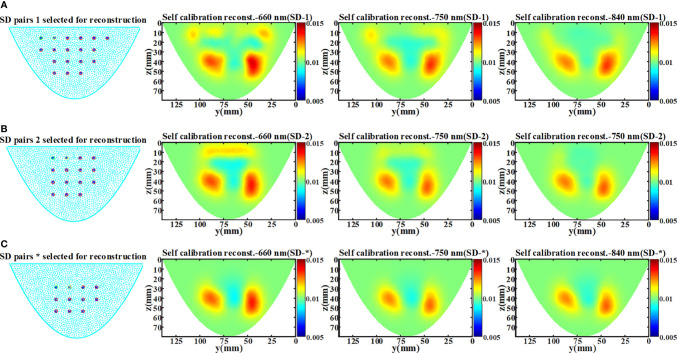
Experimental results of artifact analysis. The * represents the setting of the above phantom experiment in section 5.2. **(A)** The arrangement of 18×18 SD pairs and the reconstructed x-slice (x=15 mm) images of three wavelength (SD-1). **(B)** The arrangement of 15×15 SD pairs and the reconstructed x-slice (x=15 mm) images of three wavelength (SD-2). **(C)** The arrangement of 11×11 SD pairs and the reconstructed x-slice (x=15 mm) images of three wavelength (SD-*).

In the phantom experiment, we found artifacts in the reconstructed image recovered by the data self-calibration method. This may be due to the effect of the selection of SD measurement pairs on the accuracy of the ‘virtual reference measurement’ data estimation. When DOT measurement is performed on a homogeneous object, even at the same SD distance, the light intensity obtained by the SD pair near the object boundary and the SD pair in the central region of the object is different. Therefore, in practical applications, using the measured data of all SD pairs at the same SD distance for the estimation of ‘virtual reference measurements’ tends to hinder the performance of the proposed method. To verify that the use of SD pairs located in the boundary region may introduce artifacts in the recovered images, reconstructions were performed using three different numbers of SD pairs. As shown in **Figures 11A–C**, the artifacts in the image are significantly reduced as the SD pairs at the boundary are removed.

**Figure 12:**
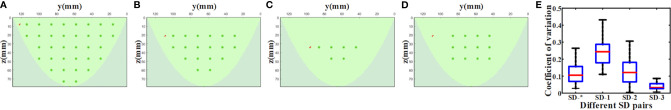
Non-boundary region analysis experiment results. Different SD pairs selected for reconstruction: **(A)** SD-1, **(B)** SD-2, **(C)** SD-3, **(D)** SD-*. **(E)** The coefficient of variation of measured data of different SD pairs with the same SD distance under the four settings.

For imaging objects of different sizes, the proper selection of the SD measurement pairs from non-boundary region is also crucial for the proposed data self-calibration method. We utilize the simulation experiments to analyze the determination of non-boundary regions, and the settings of the homogeneous phantom are the same as those in Section 4.1. Based on the arrangement of the sources and detectors of our DOT imaging system, we adopted four different SD pairs selection strategies and obtained ‘virtual reference measurement’ data using the data self-calibration method. **Figure 12A** shows all the light sources and detectors covered by the simulation phantom. **Figures 12B, C** show the measurement layout after removing the outermost circle of light sources/detectors in turn. **Figure 12D** is the arrangement of SD pairs used in this paper. For diffuse optical measurements of large-size homogeneous turbid media, we use the coefficient of variation to assess the differences among measured data of different SD pairs with the same SD distance. With the elimination of SD pairs at the boundary, the differences in the measured data of different SD pairs under the same SD distance are significantly reduced (**Figure 12E**). The coefficient of variation can be utilized to separate non-boundary regions and boundary regions, and the threshold of the coefficient of variation needs to be determined. The results in Section 5.1 show that the reconstruction quality of the 12×12 SD pairs used in this paper is equivalent to the reference measurement calibration method. In this work, we set the division threshold to 0.3 based on the SD arrangement of the imaging system, MC simulations, and phantom experiments.

In clinical trials, we have compared the reconstructed results of PET and DOT. The PET image and the DOT image reflect FDG metabolic information and concentration of hemoglobin/blood oxygen information, respectively. The reconstructed images in **Figures 6A-E** showed correspondence. In future work, we will evaluate the sensitivity and quantification of DOT and PET images in tumor diagnosis and treatment evaluation. Besides, it should be note that the method proposed in this paper is not suitable for DOT systems with a small number of different SD pairs at the same SD distance and situations where the distribution of absorption heterogeneous region is complex (e.g., small animals). It is more suitable for cases where the size of absorption homogeneous region is larger than the absorption heterogeneous region, such as the breast tissue.

## 6 Conclusion

In this paper, we propose a data self-calibration method for DOT reconstruction. Relying on the geometric symmetry of the high-density parallel plate DOT system, the reference predicted data can be estimated directly from the task measured data. The performance of the method has been validated by a series of experiments, and the results indicated that the data self-calibration method can provide a reliable and simple solution for relative measurements in breast DOT reconstruction.

## Data Availability Statement

The raw data supporting the conclusions of this article will be made available by the authors, without undue reservation.

## Ethics Statement

The studies involving human participants were reviewed and approved by Ethics Committee of Xijing Hospital. The patients/participants provided their written informed consent to participate in this study.

## Author Contributions

XW wrote the manuscript. RH did the simulation. YHW and SZ guided the work. QY tested the experimental system. YRW and FK recruited breast cancer patients. All authors contributed to the article and approved the submitted version.

## Conflict of Interest

The authors declare that the research was conducted in the absence of any commercial or financial relationships that could be construed as a potential conflict of interest.

## Publisher’s Note

All claims expressed in this article are solely those of the authors and do not necessarily represent those of their affiliated organizations, or those of the publisher, the editors and the reviewers. Any product that may be evaluated in this article, or claim that may be made by its manufacturer, is not guaranteed or endorsed by the publisher.
